# Influences of health facility type for delivery and experience of cesarean section on maternal and newborn postnatal care between birth and facility discharge in Malawi

**DOI:** 10.1186/s12913-020-4958-4

**Published:** 2020-02-24

**Authors:** Eunsoo Timothy Kim, Kavita Singh, Ilene S. Speizer, Clara Lemani

**Affiliations:** 1 Duke Global Health Institute, Duke University, Durham, NC USA; 2 Department of Maternal and Child Health, Gillings School of Global Public Health, University of North Carolina at Chapel Hill, Chapel Hill, NC USA; 3 MEASURE Evaluation/Carolina Population Center, University of North Carolina at Chapel Hill, Chapel Hill, NC USA; 4 Carolina Population Center, University of North Carolina at Chapel Hill, Chapel Hill, NC USA; 5 UNC Project – Malawi, Lilongwe, Malawi

**Keywords:** Postnatal care, Facility delivery, Quality of care, Public facilities, Cesarean section, Malawi

## Abstract

**Background:**

A number of studies in the past have looked at determinants of postnatal care. However, many of them do not distinguish between postnatal care (PNC) before discharge and after discharge for women delivering at health facilities. Conceptually and practically, factors associated with PNC before discharge and after discharge should be different. This study examines key factors for maternal and newborn PNC before discharge.

**Methods:**

Data from the 2015–16 Malawi Demographic and Health Survey were used for the study. Three categorical endogenous variables examined in the study were whether or not mothers received a postnatal check between birth and facility discharge, whether or not newborns received a postnatal check between birth and facility discharge and whether or not women delivered by cesarean section. Delivery by cesarean section was considered as a mediator in the model. The main predictor of interest was type of health facility where women delivered. Other exogenous variables included were women’s age at most recent birth, number of antenatal visits, women’s education, household wealth, parity, newborn size, region of the country and residence. Simultaneous equation modeling was used to examine the associations of interest.

**Results:**

47% of the mothers and 68% of the newborns had PNC before facility discharge. The total and direct effects of delivering in private hospitals on maternal and newborn PNC before facility discharge were significantly higher than the effects of delivering in government hospitals. The total effects of delivering in government health centers or health posts on maternal and newborn PNC before facility discharge were significantly lower than the effects of delivering in government hospitals. Delivering by cesarean section compared to delivering vaginally was positively associated with maternal and newborn PNC before facility discharge.

**Conclusion:**

It is important that all women and newborns receive PNC before they are discharged from the facility regardless of whether or not they had a complication. The same standard of quality PNC should be provided equitably across all types and affiliations of health facilities.

## Background

Over the years, there have been significant policy discussions and updates to guidelines on encouraging skilled attendance at delivery in contexts where maternal and newborn mortality is high [1]. The World Health Organization (WHO) for one has had a few changes to its policy position regarding the matter, going from supporting the training of traditional birth attendants in the 1960s, and linking them to the larger health care system to encouraging delivery by medically-trained professionals for all births today [1, 2]. This is because skilled assistance by medically trained doctors, nurses and midwives at birth could potentially prevent and manage many of the complications that would lead to mortality [3]. The evidence on this is mixed however. A meta-analysis of population-based cohort studies on the association between place of delivery and maternal and perinatal mortality in sub-Saharan Africa found that perinatal mortality was 21% higher for women delivering at home compared to those delivering at health facilities [4]. On the contrary, the opposite relationship was true for maternal mortality whereby women delivering in health facilities had poorer outcomes [4]. This may be confounded by women with higher risk of delivery complications and mortality seeking facility-based care [4]. A more recent study from Ghana found that delivering in health facilities was not necessarily associated with lower maternal and newborn mortality risks [5]. The study concluded that women should only be recommended to seek delivery care at facilities that are capable of performing comprehensive emergency obstetric care, emergency newborn care or have competent providers [5]. Regardless, this strategy has been promoted and has seen noticeable shifts in recent years [3, 6]. The percentage of births in facilities has sizably increased in developing regions globally [3], including sub-Saharan Africa even though its increase was less dramatic compared to other regions of the world [3, 7]. A recent secondary analysis of 25 sub-Saharan African countries found that increases in facility delivery over time can mostly be attributed to increases in the facility delivery in the public sector and that the gap in facility delivery between the poorest and the wealthiest also narrowed over time [6].

In Malawi, one of the poorest countries in sub-Saharan Africa, facility delivery has also risen quite significantly over the years (from 1992 to 2015–16), going from 55 to 91%, respectively [8]. Considering that facility delivery has now become much more popular in Malawi than home deliveries, 91% versus 7% respectively [8], it is important to make sure that women take full advantage of all the benefits that come with delivering at a health facility even after birth. One of the major advantages of delivering at a health facility for mothers and their newborns is the opportunity to receive timely postnatal checks between birth and facility discharge [9]. The WHO in fact recommends that women and their newborns delivered vaginally receive continuous care for at least the first 24 h after birth in the delivering health facility before discharge [9]. There are no specific guidelines on length of stay after cesarean section but women are expected to stay in the facilities longer after the procedure.

This recommendation was meant to give mothers and newborns an opportunity to be professionally checked and monitored for any potential and unexpected danger signs that might arise during this period [10]. During the first 24 h after birth, medically trained providers at the health facility are advised to give all newborns an immediate assessment of danger signs, a clinical examination at one hour of birth and another examination right before discharge [10]. For all delivering mothers, health providers are advised to check for excessive bleeding, signs of infection, uterine contraction, fundal height and any difficulty with breastfeeding [10].

A number of studies in the past have looked at determinants of postnatal care (PNC) [11–17]. However, most of these studies have used PNC within a certain time period as the outcome without making a distinction between PNC before discharge and PNC after discharge for women delivering at health facilities [11–17]. This is an important distinction to make because conceptually and practically, factors associated with PNC between birth and facility discharge may have less to do with women’s personal preferences and choices although they may control the length of stay at the facility. The care environment at the facility is possibly a stronger influence on receipt of PNC between birth and facility discharge.

This conceptualization also aligns with the Three Delays Model [18] because receipt of PNC between birth and facility discharge depends on factors pertaining to the health system which is largely outside of the women’s control. In the Three Delays Model, the first and second phases of delay pertain to barriers in deciding to seek facility-based care and in reaching facility-based care once the decision has been made [18]. The third phase of delay consists of system-level barriers where even after women reach the health facilities, they are unable to receive adequate care due to poor staffing at the facility, lack of provider skills and knowledge and low stock of essential supplies and medicines [18]. As facility delivery continues to rise and the barriers to facility PNC potentially exist in the “third phase” for this context, a proper assessment of the current situation is warranted.

This study therefore aims to address this seeming research gap by examining whether delivery in different types of health facilities is associated with maternal and newborn PNC between birth and facility discharge in Malawi. Because cesarean section is an indicator for high risk deliveries, delivery by cesarean section will be examined as a potential mediator between type of health facility where women delivered and maternal and newborn postnatal health check between birth and facility discharge. The effects of other maternal and newborn characteristics such as age at most recent birth, number of antenatal visits, household wealth, education, parity, newborn size, residence and region will also be examined in this context.

## Methods

### Data

Women’s questionnaire data from the 2015–16 Malawi Demographic and Health Survey (MDHS) were used for the study. The 2015–16 MDHS collected a wide range of information including demographic indicators, fertility and mortality measures, family planning knowledge and use, immunization coverage, maternity care, infant and young child feeding, nutritional status, HIV and more [8]. The survey was conducted with a two-stage stratified cluster sampling design [8]. Each of the 28 administrative districts in Malawi was stratified into urban and rural strata [8]. From each strata, a sample of standard enumeration areas, which consist of about 235 households on average, were selected for household listing serving as a sampling frame [8]. In the second stage, 30 households were selected from each urban household listing or cluster and 33 households were selected from each rural household listing or cluster [8]. In the selected households, women’s questionnaires were administered to women between the ages of 15 and 49 who were either residents there or were visitors from the night before the survey [8]. In the current study, women who had a facility delivery in the last five years preceding the survey were considered for analysis and the focus is on the most recent singleton birth in a facility.

### Variables

Three categorical endogenous (outcome) variables were examined in the study: whether or not mothers received a postnatal check between birth and discharge from the facility for their most recent birth in the past 5 years, whether or not their most recent newborns received a postnatal check between birth and discharge from the facility and whether or not women delivered by cesarean section. For all three endogenous variables, “1” indicated receipt of services and “0” indicated otherwise. There were no women who responded “don’t know” for maternal PNC between birth and discharge. For newborn PNC between birth and discharge, about 1% of the women responded “don’t know.” Information about how long after delivery the first check took place and the type of provider who checked on the health of the mother and newborn were not incorporated into creating the final outcome measures. This is because, as an exploratory research study, we wanted to understand and capture all postnatal checks that occur between birth and facility discharge and not just those that occur within the first 24 h. Among all women who had their most recent birth in the 5 years prior to the survey, about 7% of them had missing data for maternal and newborn PNC between birth and discharge.

Winship and Mare mention in their important work on structural equations for discrete data [19] that binary indicators can represent one of two ideas: an indicator that measures a discrete event or an indicator that serves as a proxy for an unobserved underlying continuous variable [19]. In this study, delivery by cesarean section is treated as a proxy variable for some underlying continuous phenomenon. The final decision to perform cesarean section is likely based on whether pregnant women, who are all on an unobserved continuum of complication risk, display characteristics that exceed providers’ threshold levels for risk of complication. Hence, this study acknowledges that the underlying continuous variable exists for cesarean section, albeit unobserved.

The main predictor of interest in the study was type of health facility where women delivered. It was coded to have four categories: [1] government hospital [2]; government health center, government health post and other public sector facilities (not specified in the dataset) [3]; private hospital and Christian Health Association of Malawi (CHAM) hospitals; and [4] CHAM health centers, Banja La Mtsogolo clinics (BLM) and other private sector facilities (not specified in the dataset). Health centers, health posts and other unspecified facilities were grouped together because only a small number of women delivered in health posts and other unspecified facilities (around 1.44%) and deliveries are typically done in health centers or hospitals in Malawi [20]. All of these facility categories represent the major health service providers in Malawi [20]. However, there are a couple of differences between health facilities based on their type and affiliation (public and private). Government-owned facilities provide services free of charge while privately-owned facilities charge a fee [20]. However, the possibility cannot be ruled out that even at government-owned facilities, health services may not be free at the point of use in some parts of the country. Health facilities of various types and affiliations also have very different resources for service readiness including basic amenities, equipment, infection control, diagnostic capacity, essential medicines, quality assurance, client feedback and provider training [20]. The main predictor variable was meant to capture these differences in general service readiness as well as potential differences in PNC practices by types and affiliations of health facilities.

Other exogenous variables included in the model were women’s age at most recent birth, number of antenatal visits, women’s education, household wealth, parity, newborn size, region of the country and residence. Women’s age at most recent birth was included as a continuous variable. Number of antenatal visits was coded as having had “less than 4 visits” or “4 or more visits”. Women’s education was coded to be either “no education”, “primary education” or “secondary education”. Household wealth was a quintile variable constructed by the Demographic and Health Surveys Program (DHS) using principal components analysis [8] and it was coded as either “poorest”, “poorer”, “middle”, “richer” or “richest”. Parity of the birth was coded as either “1”, “2 – 3” or “4 or more”. Newborn size based on women’s recall was coded as either “very large”, “larger than average”, “average”, “smaller than average” or “very small”. Region of the country was coded as either “northern”, “central” or “southern”. Residence was coded as either “urban” or “rural”.

In this study, receiving a postnatal check between birth and facility discharge was hypothesized to be more a function of the type of facility where women delivered and other “noticeable” maternal and newborn characteristics that could further attract attention by the providers in the respective facilities. However, sociodemographic variables such as women’s age at the time of birth and education were also included in the model to test for their effects.

Descriptive analyses of the study variables are presented in Tables 1, 2 and 3. Table 1 presents the coverage of maternal and newborn postnatal health checks between birth and facility discharge among women who had their most recent singleton birth in facilities in the 5 years prior to the survey. Table 2 presents descriptive summary information of the study variables for women who had their most recent singleton birth in facilities in the 5 years prior to the survey. Table 3 presents the percentages of women and newborns receiving postnatal health checks between birth and discharge by type of delivering health facility.

**Table 1:** Coverage of maternal and newborn postnatal health checks between birth and facility discharge, MDHS 2015–16

	Total	
	N	%
Maternal postnatal health check between birth and facility discharge		
No	6427	53%
Yes	5723	47%
Newborn postnatal health check between birth and facility discharge		
No	3882	32%
Yes	8152	68%

Note

Population-weighted counts of women who had their most recent singleton birth in facilities in the past 5 years were reported from the MDHS 2015–16

Column percentages sum to 100%

**Table 2:** Descriptive information about the study variables for women who had their most recent singleton birth in facilities in the past 5 years, MDHS 2015–16

	Total	
	N	%
Type of health facility where women delivered		
Government hospital	3689	30%
Government health center/health post/others	6783	56%
private/CHAM/mission hospital	1058	9%
CHAM health center/blm/others	621	5%
Cesarean section		
No	11352	93%
Yes	798	7%
Antenatal Visits		
Less than 4 visits	5810	48%
4 or more visits	6291	52%
Education		
No education	8219	68%
Primary education	2962	24%
Secondary education	970	8%
Household Wealth		
Poorest	2749	23%
Poorer	2594	21%
Middle	2337	19%
Richer	2235	18%
Richest	2235	18%
Parity		
1	3126	26%
2–3	4561	38%
4+	4464	37%
Residence		
Urban	1827	15%
Rural	10324	85%
Region of the country		
Northern	1432	12%
Central	5094	42%
Southern	5625	46%
Newborn size		
Very large	1166	10%
Larger than average	3142	26%
Average	5980	50%
Smaller than average	1295	11%
Very small	488	4%
	Mean	Std Error
Age at Most Recent Birth (continuous)	26.54	0.086

Note

Population-weighted counts of women who had their most recent singleton birth in facilities in the past 5 years and either received maternal postnatal health check between birth and discharge or not were reported from the MDHS 2015–16 (weighted total = 12,150)

Column percentages sum to 100%

**Table 3:** Type of health facility where women delivered stratified by maternal and newborn postnatal health check between birth and facility discharge in Malawi, MDHS 2015–16

	Maternal postnatal health check between birth and facility discharge				Newborn postnatal health check between birth and facility discharge			
	Yes		No		Yes		No	
	N	%	N	%	N	%	N	%
Type of health facility where women delivered	***				***			
Government hospital	1930	52%	1759	48%	2626	72%	1016	28%
Government health center/health post/others	2860	42%	3924	58%	4286	64%	2452	36%
private/CHAM/mission hospital	637	60%	420	40%	811	78%	234	22%
CHAM health center/blm/others	297	48%	324	52%	429	70%	181	30%

Note

***p < 0.001

Row percentages sum to 100%

The weighted totals were 12,150 for maternal postnatal health check between birth and discharge and 12,033 for newborn postnatal health check between birth and discharge (most recent singleton births in facilities in the past 5 years)

### Analysis

The model hypothesizes that: [1] cesarean section, age of the mother at most recent birth, education of the mother, parity, number of antenatal visits, type of health facility where women delivered, urban/rural residence and region of the country influence maternal postnatal health check between birth and discharge [2]; cesarean section, newborn size, education of the mother, number of antenatal visits, type of health facility where women delivered, urban/rural residence and region of the country influence newborn postnatal health check between birth and discharge; and finally, [3] all of the exogenous variables in the model, except education, influence delivery by cesarean section.

Because cesarean section is in the mediated pathway for most of the exogenous variables in the model, indirect effects of these variables on maternal and newborn postnatal health checks can be calculated. In the case where exogenous variables predict the latent continuous variable underlying the binary mediator and the same latent continuous variable is used to predict maternal and newborn PNC, the calculation of indirect effects just involves the product of two coefficients: the coefficient of an exogenous variable in predicting cesarean section and the coefficient of cesarean section in predicting maternal or newborn PNC [19]. This corresponds to the model 1 specification in the article by Winship and Mare [19]. The total effects are the sum of the direct effects and the indirect effects [19].

The indirect effects through cesarean section and the total effects were only calculated and interpreted for type of health facility where women delivered. This is because the primary interest of the study is to distinguish the direct effects of the delivering health facilities from their indirect effects through cesarean section. According to the Malawi Service Provision Assessment 2013–14 report, cesarean section is only done in hospitals [20]. However, the indirect effects and the total effects were also calculated for government and CHAM health centers because some women in the sample responded that they delivered by cesarean section in these lower-level facilities as well. The effects of other exogenous variables will indicate whether observable maternal and newborn characteristics as well as the socioeconomic background factors influence postnatal health check at the facilities outside of the mediated pathway through cesarean section.

The hypothesized model also shows that all of the exogenous variables are correlated in some way. It does not specify what the directions of the associations are among the exogenous variables. This is because those relationships are not of interest in the study. However, acknowledging that these variables are correlated is important for model specification and fit. Lastly, the residuals of maternal postnatal health check between birth and discharge and newborn postnatal health check between birth and discharge are hypothesized to correlate in the model. Due to this correlation, the model is not “fully recursive”. However, it is still “recursive” because there are no feedback relationships present in the model.

### Checks for model identification and specification

**Fig. 1:**
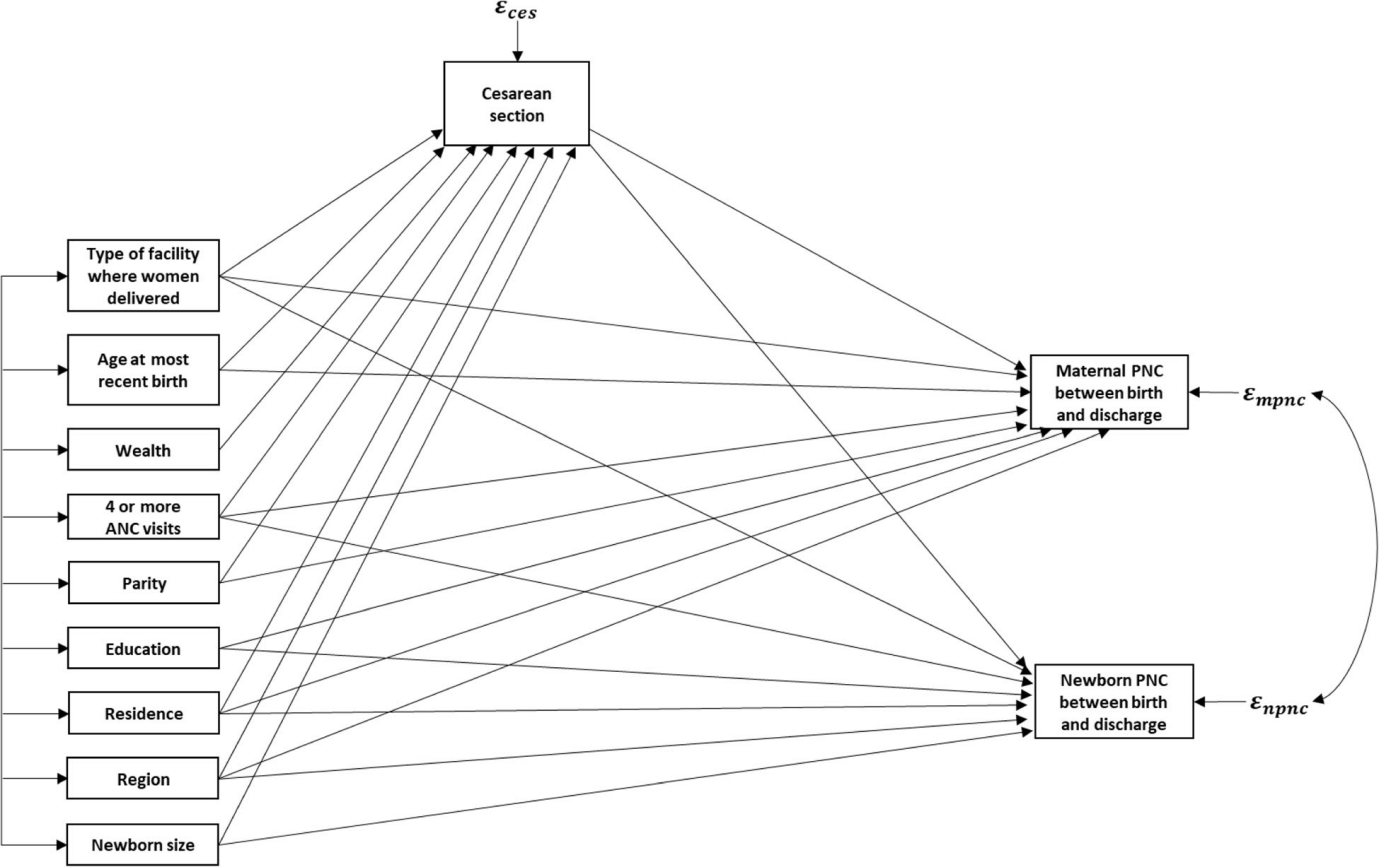
Path diagram of the hypothesized model

There are many rules of identification reported in the literature to help researchers make sure that their models are identified. One of the rules of identification that applies to recursive models with correlated errors is that no variable influences another variable with the error terms of each being correlated [21]. According to Brito and Pearl, this is a sufficient condition of identification for recursive models with correlated errors [21]. The illustrated path diagram in Fig. 1 shows that this rule applies and is sufficient for identification.

Several model fit indices with the full information estimation method showed that the model specification fit the data well. Specifically, six different model fit indices were considered to assess if the model specification was appropriately fitting the data. These indices were the overall chi-square test, Tucker-Lewis index, incremental fit index, relative noncentrality index, root mean square error of approximation and the Bayesian information criterion [22, 23]. All of the six model fit indices suggested that the current model specification is consistent with the data and that no other modifications need to be made based solely on model fit. The summary of the model fit indices can be found in Table 4.

**Table 4:** Tests for model specification

	Test results	Indication of good model fit
Diagonally-weighted least squares chi-square test		
Test statistic	15.312	
Degrees of freedom	17	*P* - value > 0.05
*P* - value	0.573	
Tucker-Lewis Index (TLI)	1.000	TLI = 1 is ideal; TLI > 0.95 is acceptable
Incremental Fit Index (IFI)	1.000	IFI = 1 is ideal; IFI > 0.95 is acceptable
Relative Noncentrality Index (RNI)	1.000	RNI = 1 is ideal; RNI > 0.95 is acceptable
Root Mean Square Error of Approximation (RMSEA)	0.000	RMSEA = 0 is ideal; RMSEA < 0.06 is acceptable
BIC test	− 144.301	BIC < 0

Table 5 presents probit regression coefficients from the simultaneous equation model that were estimated using the “lavaan” package in R studio. The probit regression coefficients were adjusted for cluster sampling. The signs of the probit regression coefficients can be interpreted as positive or negative influences on the binary outcomes themselves and on their underlying continuous variables. Lastly, because all endogenous variables in the model are categorical, diagonally-weighted least squares were used.

**Table 5:** The total, direct and indirect effects of key predictors on maternal and newborn postnatal health checks between birth and facility discharge in Malawi, MDHS 2015–16

	Maternal postnatal health check between birth and facility discharge		Newborn postnatal health check between birth and facility discharge	
	Coef	Std Error	Coef	Std Error
Type of health facility where women delivered				
*Total Effects:*				
Government hospital (ref)	–	–	–	–
Government health center/health post/others	−0.147***	0.027	−0.127***	0.028
private/CHAM/mission hospital	0.127**	0.044	0.161**	0.049
CHAM health center/blm/others	−0.013	0.055	0.031	0.059
*Direct Effects:*				
Government hospital (ref)	–	–	–	–
Government health center/health post/others	0.156***	0.038	0.021	0.041
private/CHAM/mission hospital	0.184***	0.045	0.189***	0.049
CHAM health center/blm/others	0.218**	0.064	0.143*	0.065
*Indirect Effects (*via *cesarean section):*				
Government hospital (ref)	–	–	–	–
Government health center/health post/others	−0.304***	0.028	−0.147***	0.028
private/CHAM/mission hospital	−0.057**	0.018	−0.028**	0.010
CHAM health center/blm/others	−0.231***	0.038	−0.112***	0.026
Cesarean section				
No (ref)	–	–	–	–
Yes	0.294***	0.023	0.143***	0.026
Age at Most Recent Birth (continuous)	−0.000	0.003	NA	NA
Antenatal Visits				
Less than 4 visits (ref)	–	–	–	–
4 or more visits	0.080**	0.025	0.076**	0.025
Education				
No education (ref)	–	–	–	–
Primary education	0.080**	0.029	0.059	0.031
Secondary education	0.277***	0.051	0.163**	0.056
Parity				
1 (ref)	–	–	–	–
2–3	0.058	0.034	NA	NA
4+	0.121*	0.050	NA	NA
Residence				
Urban (ref)	–	–	–	–
Rural	−0.181***	0.039	−0.217***	0.041
Region of the country				
Northern	0.240***	0.036	0.452***	0.039
Central (ref)	–	–	–	–
Southern	0.034	0.028	−0.002	0.027
Newborn size				
Very large	NA	NA	0.023	0.043
Larger than average	NA	NA	0.017	0.030
Average (ref)	–	–	–	–
Smaller than average	NA	NA	0.002	0.039
Very small	NA	NA	0.095	0.064
	Estimate		Std Error	
Covariance of the errors of maternal postnatal health check and newborn postnatal health check	0.717***		0.013	
	R-square estimates		Thresholds	
Maternal postnatal health check	0.135		0.318***	
Newborn postnatal health check	0.088		−0.344***	
Cesarean section	0.260		1.805***	

Note

*p < 0.05 **p < 0.01 ***p < 0.001

There were 11,956 observations used for the analysis (most recent singleton births in facilities in the past 5 years)

Probit regression coefficients and standard errors of the study variables are presented. The estimates were obtained in R studio with the lavaan package

Total effects were calculated as the sum of the direct effects and the indirect effects

“NA” stands for not applicable. The corresponding variables were not included in the model for the particular outcome being predicted and therefore, no estimates were obtained

## Results

Among women who had their most recent singleton birth in facilities in the past 5 years preceding the survey, 47% had a maternal postnatal health check between birth and facility discharge and 68% had a newborn postnatal health check between birth and facility discharge (See Table 1). The majority of women delivering in facilities either delivered in government hospitals (30%) or government health centers and health posts (56%). Although not shown in the tables, very few women actually delivered in government health posts and other public facilities (around 2%). Delivery in private health facilities was also low with 9% delivering in private hospitals and 5% delivering in private health centers, BLM and others. Also, about 7% delivered by cesarean section and about half of the newborns were either in the large or small categories (combined 51%). A combined 14% of the newborns were either in the very large or very small categories.

An average woman who had her most recent singleton birth in a health facility in the past 5 years preceding the survey was about 27 years old. In addition, the majority of women had two or more births (75%), lived in rural areas (85%) and were from the central and southern regions of Malawi (88%). As for education, 68% of women had no education and 24% had primary education. 44% of women belonged in either the “poorest” or “poorer” wealth quintiles and 36% of women belonged in the “richer” or “richest” wealth quintiles. Slightly more than half of the women attended four or more antenatal visits (52%) (See Table 2).

Among women delivering in government hospitals, 52% received a maternal postnatal health check between birth and discharge and 72% received a newborn postnatal health check between birth and discharge. Among women delivering in government health centers, government health posts or other public facilities, 42% received a maternal postnatal health check between birth and discharge and 64% received a newborn postnatal health check between birth and discharge. For women delivering in private, CHAM or mission hospitals, 60% received a maternal postnatal health check between birth and discharge and 78% received a newborn postnatal health check between birth and discharge. Lastly, among deliveries in CHAM health centers, BLM and others, 48% received a maternal postnatal health check between birth and discharge and 70% received a newborn postnatal health check between birth and discharge (See Table 3).

For the main predictor of interest, type of health facility where women delivered, the direct effects, the indirect effects via cesarean section and the total combined effects were examined separately. Delivering in a government hospital was the referent category to which deliveries in all other health facilities were compared. In comparison to the direct effect of delivering in a government hospital, the direct effect of delivering in a government health center, health post or others was significantly higher on maternal postnatal health check between birth and discharge and not significant on newborn postnatal health check between birth and discharge. The direct effects of delivering in any type of private facility were significantly higher on both maternal and newborn postnatal health checks between birth and discharge compared to the direct effect of delivering in a government hospital. As for the indirect effects via cesarean section, the effects of delivering in government health centers, government health posts, private, CHAM or mission hospitals, CHAM health centers, BLM or others were all significantly lower on maternal and newborn postnatal health checks between birth and discharge compared to the effect of delivering in a government hospital.

For the total combined effects, delivering in government health centers, health posts and others had significantly lower effects on both maternal and newborn postnatal health checks between birth and discharge compared to the effect of delivering in a government hospital. The total combined effects of delivering in private, CHAM or mission hospitals were significantly higher on maternal and newborn postnatal health checks between birth and discharge compared to the total combined effect of delivering in a government hospital.

A few other predictors in the model also showed statistically significant effects. The effects of delivering by cesarean section on maternal and newborn postnatal health checks between birth and discharge were significantly higher compared to the effects of delivering vaginally. The effect of having attended four or more antenatal visits compared to having attended less than four antenatal visits was significantly higher on maternal and newborn postnatal health checks between birth and discharge. The effect of having had four or more births compared to having had one birth was significantly higher on maternal postnatal check between birth and discharge. The effects of living in a rural area on maternal and newborn postnatal health checks between birth and discharge were significantly lower compared to the effects of living in an urban area. In addition, the effects of living in a northern region of the country on maternal and newborn postnatal health checks between birth and discharge were significantly higher compared to the effects of living in a central region.

As for education, the effects of having had secondary education on maternal and newborn postnatal checks between birth and discharge were significantly higher compared to the effects of having had no education. The effect of having had primary education was only significant for maternal postnatal check between birth and discharge. The effects of age and newborn size were not statistically significant. Lastly, the correlation between the errors of maternal and newborn postnatal health checks between birth and discharge was high and statistically significant (See Table 5).

## Discussion

In Malawi, there has been a major shift in the trend of where women deliver their babies [8]. In the past few decades, the percentage of women delivering in health facilities has sharply increased to the point where 9 out of 10 women now deliver in health facilities [8]. This is a promising trend as it implies that women can be expected to receive skilled maternity services during delivery and immediately after birth. The WHO recommends that mothers and newborns delivering at the health facilities receive a timely postnatal health check before they are discharged to return home [9]. Considering that over 90% of Malawian pregnant women now deliver in health facilities [8], the general population of new mothers would receive at least one professional health check after birth if all health facilities followed WHO guidelines for PNC. Findings of this study indicate that this is not the case.

Across all public and private health facilities, the percentage of women receiving a postnatal health check between birth and discharge ranged between a low of 42% and a high of 60%. The percentage of newborns receiving a postnatal health check between birth and discharge ranged between 64 and 78%. Among mothers and newborns who received a postnatal health check between birth and discharge, around 90% received a check within the first day of delivery which is encouraging (not shown in tables). A lower percentage of mothers received a postnatal health check compared to their newborns. This could perhaps be an indication that mothers compared to their newborns tend to be neglected for care after delivery, which calls for further investigation. Aside from the fact that mothers receive less attention at health facilities in the postnatal period compared to the attention that newborns receive, the overall percentages showed that too many mothers and newborns are being missed before discharge across the board. This is a concern especially for government health facilities as the majority of Malawian women who deliver in facilities used either government hospitals or government health centers. A relatively small percentage of women used private services. This implies that government-affiliated health facilities should be the main target for interventions in order to have a wide coverage of pregnant women and newborns. The primary focus should be on ensuring that all women and newborns receive a postnatal health check before leaving the facilities in which they delivered.

Findings from the simultaneous equation model corroborate the need for such an intervention focus. We found that women delivering in private, CHAM or mission hospitals had significantly higher direct and total effects on both maternal and newborn postnatal checks between birth and discharge compared to the effects of delivering in government hospitals. We also found that delivering in government health centers, health posts and others had significantly lower total effects on maternal and newborn postnatal checks between birth and discharge compared to the effects of delivering in government hospitals. These findings could potentially lend support to an argument that the amount of postnatal attention women and newborns receive may depend on the established practices and protocols that are unique to the type of health facility where women deliver. It is also likely that varying patient volume affects the quality of care that patients receive at different types of facilities. Regardless of the cause, such pattern of observation is concerning as the same standard of PNC should apply to all women and newborns without any conditions.

Kruk et al., in their recent study examining quality of basic maternal care functions in health facilities of five African countries (Kenya, Namibia, Rwanda, Tanzania and Uganda), found that quality of basic maternal care functions was significantly lower for primary care facilities compared to secondary care facilities [24]. In addition, for both primary care and secondary care facilities, private facilities had significantly higher quality of basic maternal care functions compared to public facilities [24]. Another finding of importance was that low volume of delivery was negatively associated with quality of basic maternal care functions for both primary care and secondary care facilities [24]. The authors speculated that in an environment where there is low frequency of delivery and complications, providers may have difficulty retaining necessary clinical skills [24]. Quality of basic maternal care functions was constructed as an index of 12 items including those related to facility infrastructure and care practices for normal and emergency situations [24]. Although Malawi was not one of the countries examined in the study, it may exhibit similar facility care patterns that were described in the study as these countries are clustered in a similar geographic region.

On average, receiving cesarean section during delivery was also positively associated with maternal and newborn postnatal health checks between birth and discharge, which again calls into question whether the same standard of PNC is being applied across the health facilities, mainly hospitals and a few health centers, that are performing cesarean section. This calls for a re-examination of PNC strategies and protocols at the health facility level. It would also be important to make sure that providers do not consider PNC as only necessary when there is a complication. According to the Malawi Service Provision Assessment 2013–14 report, about 60% of surveyed health providers in hospitals and health centers in Malawi indicated that they received in-service training related to delivery and newborn care [20]. In clinics, 51% received this type of training [20]. Despite evidence that general in-service trainings are provided across different levels of health facilities in Malawi [20], more targeted and specific trainings on providing universal and quality preventative PNC may be necessary.

In addition, the issues of facility under-staffing [25], over-burdening of the providers by existing workloads [25] and demotivation by the lack of organizational-level accountability, support and incentive structures [26] need to be addressed. These factors likely contribute to shortened length of stay at the facility after delivery, which in turn, could lead to less mothers and newborns being checked before discharge due to time and space constraints. Hence, as a long-term strategy, it is pivotal for facilities and district managements to reform staff accountability and incentive structures so that the fundamental concerns related to human resources can be addressed.

As for the indirect effects via cesarean section, they were all significant and negative. The negative coefficients were because the effects of cesarean section on maternal and newborn postnatal checks between birth and discharge were positive, but the effects of delivering in government health centers, government health posts and private health facilities on receipt of cesarean section were significantly lower than the effects of delivering in government hospitals (See Additional file 1). This is reasonable as cesarean section is primarily done in hospital settings and not in health centers or health posts in Malawi [20]. The percentage of women reporting to have delivered by cesarean section in either health centers or health posts was only 12.6% (not shown in tables).

It is also important to note that women’s age and newborn size did not have statistically significant effects, with cesarean section specified as a mediated pathway in the model. This could perhaps suggest that more than these maternal and newborn characteristics themselves, receipt of cesarean section was a stronger predictor of whether women and newborns received postnatal health checks between birth and discharge.

Interestingly, the effects of having had four or more antenatal visits and the effects of having had secondary education were significantly positive for maternal and newborn postnatal checks between birth and discharge. These findings are not intuitive to understand as receipt of maternal and newborn postnatal checks between birth and facility discharge should theoretically be a function of the care practices at the health facilities and not characteristics of the patients. The findings suggest, however, that women’s level of education and their frequency of antenatal visits do play a part in whether mothers and newborns receive postnatal checks between birth and facility discharge. It is possible that more educated women and women who are better informed of the importance of PNC through their past antenatal visits, ask to be checked before discharge if the facility providers do not seem to initiate. However, this is only speculation and further research should look into the reasons why this may be the case.

Delivering at a health facility has clear advantages over delivering at home in low-resource settings [27]. One of the main advantages is that being in a health facility gives women and newborns an opportunity to receive skilled care during delivery and be professionally examined for any unexpected danger signs that might arise in the immediate postnatal period [27]. However, it is a fallacy to assume that increases in facility delivery by default correlate with increases in coverage of timely PNC for both mothers and newborns. Increases in the public’s demand for facility delivery should be met with provision of quality health services not only for delivery but also for the time period following immediately afterwards. Preventative health services with life-saving potential should also be provided equitably across all types and levels of health facilities, both public and private. Women and their newborns should not be receiving inconsistent quality and standards of PNC because they chose or happened to deliver in a certain type of health facility.

Special attention should also be given to rural areas of Malawi as living in rural areas was negatively associated with both maternal and newborn postnatal health checks between birth and discharge compared to living in urban areas. The key objective is to encourage all health facilities to provide quality preventative postnatal health checks to all delivering women and their newborns before discharge as a regular protocol no matter the patients’ actual or perceived need of greater care.

This study has several limitations. A noteworthy limitation is that delivery by cesarean section may not be a great proxy measure of complicated cases or high-risk scenarios as the likelihood of receiving cesarean section is systematically different based on the type of health facility where women delivered. In other words, complicated deliveries in health centers or clinics may not have been well represented by the proxy measure compared to the complicated deliveries in hospitals. Second, individual R-square values for the three equations were not too high. This could perhaps suggest that receipt of maternal and newborn postnatal health checks at the delivering health facility are influenced by more detailed facility-level and provider-level characteristics that are unobserved. It is also likely that having a measure for women’s knowledge regarding the importance of PNC could help. Third, some of the study variables, including postnatal checks between birth and discharge, are subject to potential recall bias and misreport [28]. However, a recent validation study from Kenya and Swaziland found that women’s self-reports for many of the postnatal care indicators matched the trained personnel’s direct observation [29]. Fourth, by only including women’s most recent birth in the past 5 years preceding the survey, there is a possibility that older women and women with higher fertility are underrepresented because they are less likely to have given birth in the past 5 years. Fifth, there could be other potential mediators between the type of health facility where women delivered and maternal and newborn postnatal checks between birth and discharge. Some of these variables include length of stay at the facility and immediate breastfeeding among others. Exclusion of such variables in the model (in order to keep the model parsimonious for the main research questions) means that the direct effects of the type of health facility where women delivered on maternal and newborn postnatal checks are really the total effects that incorporate the omitted pathways through these potential mediators. Lastly, the effects reported in this study are associations rather than causal relationships and therefore should be interpreted with caution. Future research should explore these areas further and improve upon these limitations.

## Conclusions

The trend has shifted towards facility delivery in Malawi [8]. This has significant public health implications for women and newborns as being in the facility presents an opportunity to receive timely and skilled PNC. Health facilities should revisit their current PNC strategies and protocols to examine if providers are following WHO guidelines [9]. Addressing more fundamental problems related to human resources should also be considered. The goal is to have all women and newborns delivering at the facilities checked without conditionality before they are discharged.

## Supplementary information

**Additional file 1:** Appendix Table. The effects of key predictors on receipt of cesarean section in Malawi, MDHS 2015–16.

## Data Availability

The dataset analyzed during the current study is available upon request from the official DHS website.

## Abbreviations

BLM: Banja La Mtsogolo Clinics
CHAM: Christian Health Association of Malawi
DHS: Demographic and Health Surveys
MDHS: Malawi Demographic and Health Survey
PNC: Postnatal Care
WHO: World Health Organization
