# Supplementary material for: Influences of health facility type for delivery and experience of cesarean section on maternal and newborn postnatal care between birth and facility discharge in Malawi
Source: BMC Health Serv Res. 2020 Feb 24;20:139. doi: 10.1186/s12913-020-4958-4 (PMC7041203; doi:10.1186/s12913-020-4958-4)
Supplement: Supplementary file 1 — Additional file 1: Appendix Table. The effects of key predictors on receipt of cesarean section in Malawi, MDHS 2015–16. [file 12913_2020_4958_MOESM1_ESM.docx]

APPENDIX

| **Appendix. The effects of key predictors on receipt of cesarean section in Malawi, MDHS 2015-16** | | |
| --- | --- | --- |
|  | Receipt of  cesarean section | |
|  | Coef | Std Error |
| Type of health facility where women delivered |  |  |
| Government hospital (ref) | - | - |
| Government health center/health post/others | -1.034*** | 0.051 |
| private/CHAM/mission hospital | -0.194** | 0.060 |
| CHAM health center/blm/others | -0.787*** | 0.113 |
| Age at Most Recent Birth (continuous) | 0.022*** | 0.004 |
| Antenatal Visits |  |  |
| Less than 4 visits (ref) | - | - |
| 4 or more visits | 0.111** | 0.040 |
| Household wealth |  |  |
| Poorest | 0.027 | 0.066 |
| Poorer | -0.031 | 0.066 |
| Middle (ref) | - | - |
| Richer | 0.136* | 0.063 |
| Richest | 0.140* | 0.071 |
| Parity |  |  |
| 1 (ref) | - | - |
| 2-3 | -0.244*** | 0.051 |
| 4+ | -0.643*** | 0.079 |
| Residence |  |  |
| Urban (ref) | - | - |
| Rural | 0.049 | 0.055 |
| Region of the country |  |  |
| Northern | 0.210*** | 0.052 |
| Central (ref) | - | - |
| Southern | -0.034 | 0.046 |
| Newborn size |  |  |
| Very large | 0.347*** | 0.063 |
| Larger than average | 0.278*** | 0.044 |
| Average (ref) | - | - |
| Smaller than average | 0.041 | 0.065 |
| Very small | 0.011 | 0.097 |
| Note. | | |
| *p<0.05 **p<0.01 ***p<0.001 | | |
| There were 11,956 observations used for the analysis (with most recent singleton births in facilities in the past 5 years). For more detail on the model, see Table 5. | | |
| Probit regression coefficients and standard errors of the study variables are presented. The estimates were obtained in R studio with the lavaan package. | | |
